# Cadmium, Copper, Lead, and Zinc Contents of Fish Marketed in NW Mexico

**DOI:** 10.1155/2014/546897

**Published:** 2014-01-12

**Authors:** Martín G. Frías-Espericueta, Francia K. G. Zamora-Sarabia, J. Isidro Osuna-López, María D. Muy-Rangel, Werner Rubio-Carrasco, Marisela Aguilar-Juárez, Domenico Voltolina

**Affiliations:** ^1^Facultad de Ciencias del Mar, Universidad Autónoma de Sinaloa, P.O. Box 1132, 82000 Mazatlán, SIN, Mexico; ^2^Centro de Investigación en Alimentación y Desarrollo, Unidad Culiacán, 80129 Culiacán, SIN, Mexico; ^3^Centro de Investigaciones Biológicas del Noroeste, Laboratorio de Estudios Ambientales UAS-CIBNOR, P.O. Box 1132, 82000 Mazatlán, SIN, Mexico

## Abstract

To assess if they were within the safety limits for human consumption, the Cd, Cu, Pb, and Zn contents of fish muscles, bought from separate stalls of the fish markets of nine cities of NW Mexico, were determined by atomic absorption spectrophotometry. Considering all fish and markets, the mean contents were Zn: 23.23 ± 5.83, Cu: 1.72 ± 0.63, Cd: 0.27 ± 0.07, and Pb: 0.09 ± 0.04 *µ*g/g (dry weight). Cu, Zn, and Pb did not reach levels of concern for human consumption, but the high Cd values determined in Mazatlán (*Mugil cephalus*: 0.48 ± 0.15; *Diapterus* spp.: 0.57 ± 0.33; *Lutjanus* spp.: 0.72 ± 0.12; small shark: 0.87 ± 0.19 *µ*g/g dry weight) indicate that this was the only metal of concern for human health because the daily individual consumption of fish muscle to reach the PTDI would be within 0.27 and 0.41 kg.

## 1. Introduction

Fish is an important protein and essential nutrient source but, because of metal accumulation [1], fish consumption has been associated with real or potential health effects in children and adults [2].

Agriculture, mining, and food processing are the main industrial activities in the Mexican NW, and their effluents may increase the level of coastal pollution, which could be one of the reasons for the high Pb and possibly Cd levels found in aquatic organisms of Sinaloa State coastal waters [3].

There is information on the metal content of some fish species of several lagoons and coastal areas of NW Mexico [3, 4], but none is available for fish sold in public markets. In this work, we determined the cadmium (Cd), copper (Cu), lead (Pb), and zinc (Zn) contents in samples of four fish species which were selected because of their local origin and their high consumption, in view of their affordable price. Samples were bought from public fish markets of nine cities of NW Mexico to assess whether their concentrations were acceptable for human consumption.

## 2. Material and Methods

**Figure 1:**
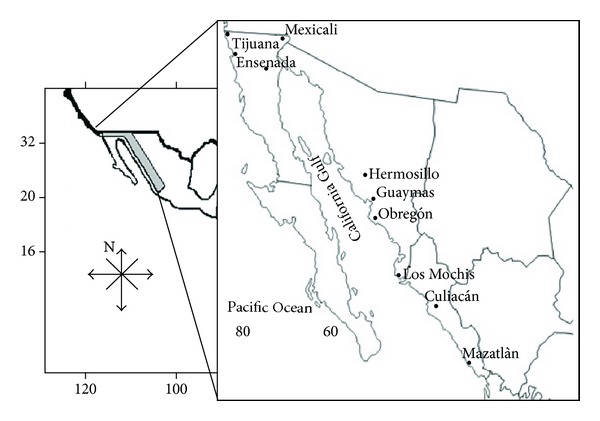
Location of the nine cities visited for sampling.

The four species selected are all landed by the artisanal fishing fleets and may therefore be considered as originating from local fishing grounds. Specimens of *Lutjanus *spp., *Mugil cephalus*, *Diapterus* spp., and of a headless, gutted, and skinned small shark (SS), probably *Rhizoprionodon longurio* which is the most common small shark caught in local fisheries [8], were obtained between February and March 2011 from the main fish markets of nine cities of NW Mexico (State of Baja California: Ensenada, Tijuana, Mexicali; Hermosillo, Guaymas, Ciudad Obregón: State of Sonora, and Los Mochis, Culiacán and Mazatlán: State of Sinaloa) (Figure 1).

In each market, the fillets of three specimens (fresh or ice-stored, depending on markets) of each species, obtained at three separate stands, were placed in separate metal-free containers and transported in an ice box to the laboratory at 4°C. All specimens of the same species obtained in the same city were homogenized, freeze-dried, and ground in a teflon mortar to obtain a composite sample.

Subsamples (0.75 g) were placed in triplicate 30 mL Teflon vessels with 5 mL of trace metal grade HNO_3_ : HCl 3 : 1 (v/v) acid mixture, digested at 130°C on a Mod block unit, transferred to clean polypropylene vials, and diluted to 25 mL with Milli-Q water [9], and the metal contents of triplicate subsamples were quantified by flame atomic absorption spectrophotometry. The presence of possible contaminants was determined with one blank every 10 subsamples with the same procedure, and the accuracy of the method was assessed with certified reference material (DOLT-4, dogfish), with percentages of recovery ranging from 91.5 to 105.6%. All materials for sampling and metal analysis were acid-washed [10].

Since the three cities of each state share the same fish sources (Pacific and Northwestern Gulf of California fishing grounds for the state of Baja California, the artisanal fisheries centered around the fishing grounds of the NE Gulf for Sonora State, and the coastal areas of the mid- and southern parts of the Gulf in the case of Sinaloa State), the mean metal contents of each of the four species obtained from the threes markets of each state were compared with one-way ANOVA tests, parametric or nonparametric, depending on the results of Kolmogorov-Smirnov's and Bartlett's tests, and the different mean values were separated with Student-Newman-Keuls tests. In all cases, the level of significance was *α* = 0.05 [11].

## 3. Results and Discussion

Considering all species and markets, the mean metal concentrations were Zn: 23.23 ± 5.83, Cu: 1.72 ± 0.63, Cd: 0.27 ± 0.07, and Pb: 0.09 ± 0.04 *µ*g/g (dry weight). For all species, the highest Cd values were in Mazatlán (*M. cephalus*: 0.48 ± 0.15; *Diapterus* spp.: 0.57 ± 0.33; *Lutjanus *spp.: 0.72 ± 0.12, and SS: 0.87 ± 0.19 *µ*g g^−1^), while the lowest were found in Tijuana (*M. cephalus* and *Lutjanus* spp.: 0.05 ± 0.07 and 0.03 ± 0.02 *µ*g g^−1^, resp.), and Culiacán (*Diapterus* spp. and SS: 0.04 ± 0.05 and 0.02 ± 0.01 *µ*g g^−1^) in the order.

The highest mean values of Cd were determined in Sinaloa. This is in agreement with the high Cd levels found in the oysters of seven coastal lagoons of this state, which might be explained by the approximately 1.5 million ha of intensive agriculture of Sinaloa state, in view of the close association between Cd- and phosphate-based fertilizers [12].

The Pb contents of *M. cephalus* obtained in Mazatlán, Hermosillo, Mexicali, and Ensenada, *Diapterus* spp. of Mazatlán, Culiacán, Obregón, Hermosillo, Guaymas, and Mexicali, and of the SS samples of Mazatlán, Culiacán, Guaymas, Mexicali, and Ensenada were below the detection limit (0.01 *µ*g g^−1^). The highest Pb values for *M. cephalus* and *Lutjanus *spp. (0.23 ± 0.09 and 0.23 ± 0.03 *µ*g g^−1^) were determined in Guaymas, whereas those of SS (0.29 ± 0.09 *µ*g g^−1^) and of *Diapterus* spp. (0.15 ± 0.12 *µ*g g^−1^) were found in Ensenada and Tijuana, respectively.

Among the metals determined in this study, Pb had the lowest values, possibly because it has low potential for bioaccumulation and biomagnification in fish [9], and also because it tends to be stored in hard calcareous structures, rather than in soft tissues [7].

Zinc ranges were 101.2–11.8, 33.5–10.3, 42.2–10.6, and 60.2–10.7 *µ*g g^−1^ for *M. cephalus*, *Diapterus* spp., *Lutjanus *spp., and SS, respectively, and the ranges for Cu were 3.55–1.79, 1.85–0.20, 5.22–0.32, and 3.72–0.37 *µ*g g^−1^ in the same order. The Cu content of *Diapterus* spp. was significantly lower in Sinaloa than in Sonora and BC (Table 1).

The highest and lowest Cu values were found in *M. cephalus* and *Diapterus* spp., respectively, and *Lutjanus* spp. and SS had intermediate mean Cu contents. There were no significant differences in the contents of the other three metals, and there were no clear relations between the mean metal contents and the trophic level (troph: [13]) of each species (Table 2).

There was no evidence of biomagnification: the tendency to a higher Cd content in SS, which has the highest trophic level (troph > 4), was not significant, and the essential Cu and Zn tended to be higher in the species with the lowest troph value.

While the mean Cd content of *M. cephalus *obtained in the Sinaloa markets is higher than most data obtained in previous studies which used this species as monitoring organism (Table 3), those of Pb are lower by at least one order of magnitude, and Cu and Zn have no clear trend. Therefore, there is no indication of metal enrichment in Sinaloa coastal waters.

Similar studies in other geographic areas show wide discrepancies as well as similarities to our results: while the Cd and Zn contents of six fish species marketed in Kayseri, Turkey (recalculated from [14]), ranged from values comparable to our data (Zn) to one order of magnitude higher (Cd: 4.9 to 10.4 *µ*g g^−1^; Zn: 78.4 to 319 *µ*g g^−1^), samples obtained off the New Jersey and the Catalonia coasts [1, 15] had Cd contents one order of magnitude lower (from 0.02 to 0.12 *µ*g g^−1^ dw, resp.), while those of Pb (0.08 and 0.52 *µ*g g^−1^) were comparable or higher than those of this study.

The provisional tolerable daily intakes (PTDI) of Cd and Cu are 58.3 [16] and 245 *µ*g person^−1^ [17]. The respective values for Pb and Zn are 10,000 and 40,000 *µ*g person^−1^ [18]. Accordingly, Cd is of possible concern for human health if bought from the fish markets of Sinaloa State, since the daily consumption to reach the PTDI would be in the order of 0.27–0.41 kg (wet weight). The nominal mean individual fish consumption in Mexico is about 11.8 kg year^−1^ [19], and for this reason it would seem that the general level of risk is probably low, although care should be taken in Sinaloa State, especially for fishermen and frequent consumers of species at the lower end of market values, such as *M. cephalus* and possibly *Diapterus* spp.

However, according to the latest national census, less than 23% of Mexican households consumed fish at least once in the preceding quarter, while fish consumption was more frequent in the 30% households of higher income, whose members would seem therefore the population at risk.

## 4. Conclusions

The public faces conflicting reports on the advantages of fish consumption, which is considered advisable for a healthy diet and for cardiovascular health [20, 21], although consumers are warned of the risks of excessive consumption, because of the heavy metals content of some fish, which casts doubts on the role of fish for a healthy diet [2, 15]. In Mexico, there are few examples of concentrations reaching levels of concern in fish caught in Pacific coastal waters. This is, however, the first report of levels of concern for the metal content of fish sold in Mexican public markets.

## Figures and Tables

**Table 1:** Mean (±standard deviation) of the metal concentrations (*µ*g g^−1^, dry weight) in edible muscle of fishes from markets of NW Mexico (one composite sample/market: *n* = 3 for each state).

Species	State	Cd	Pb*	Zn*	Cu
*M. cephalus *	Sinaloa	0.36 ± 0.01^a^	0.11 ± 0.09^a^	19.7 ± 12.7^a^	2.40 ± 0.10^a^
	Sonora	0.25 ± 0.11^a^	0.17 ± 0.14^a^	26.2 ± 12.2^a^	2.44 ± 0.73^a^
	BC	0.16 ± 0.10^a^	0.07 ± 0.10^a^	49.8 ± 44.5^a^	2.75 ± 0.66^a^

*Diapterus* spp.	Sinaloa	0.28 ± 0.27^a^	0.04 ± 0.05^a^	22.5 ± 9.5^a^	0.22 ± 0.12^a^
	Sonora	0.19 ± 0.14^a^	0.01 ± 0.01^a^	20.7 ± 3.8^a^	1.34 ± 0.32^b^
	BC	0.08 ± 0.02^a^	0.08 ± 0.07^a^	21.2 ± 10.5^a^	1.57 ± 0.24^b^

*Lutjanus* spp.	Sinaloa	0.46 ± 0.34^a^	0.12 ± 0.01^a^	20.5 ± 6.5^a^	1.99 ± 2.80^a^
	Sonora	NA	NA	NA	NA
	BC	0.17 ± 0.15^a^	0.14 ± 0.07^a^	13.4 ± 4.4^a^	1.62 ± 0.69^a^

Shark	Sinaloa	0.44 ± 0.15^a^	0.03 ± 0.04^a^	29.7 ± 26.4^a^	1.35 ± 0.84^a^
	Sonora	0.39 ± 0.17^a^	0.12 ± 0.11^a^	16.4 ± 2.3^a^	2.27 ± 1.26^a^
	BC	0.18 ± 0.08^a^	0.08 ± 0.13^a^	12.7 ± 1.8^a^	0.89 ± 0.51^a^

Different letters indicate significant difference (a < b) between the values found in the same species obtained in the markets of different states (one-way ANOVA, *α* = 0.05). *Nonparametric test, NA: data not available.

**Table 2:** Trophic level (Troph) and mean metal concentrations (*µ*g g^−1^, dry weight) in edible muscle of fishes from markets of NW Mexico.

Troph	2.1	3.3	4.0	4.2
Metal	*Mugil cephalus *	*Diapterus* spp.	*Lutjanus* spp.	Shark
Cu	2.53 ± 0.72^a^	1.04 ± 0.66^b^	1.83 ± 1.61^ab^	1.50 ± 1.01^ab^
Zn*	31.89 ± 27.58^a^	21.49 ± 7.39^a^	19.93 ± 10.45^a^	19.62 ± 15.36^a^
Cd	0.26 ± 0.13^a^	0.18 ± 0.17^a^	0.29 ± 0.25^a^	0.34 ± 0.26^a^
Pb*	0.12 ± 0.11^a^	0.04 ± 0.05^a^	0.14 ± 0.05^a^	0.08 ± 0.09^a^

Equal or common letters indicate lack of significant difference between data in the same row (one-way ANOVA, *α* = 0.05). a ≤ ab ≤ b and a < b. *Nonparametric test.

**Table 3:** Comparison of the metal contents (*µ*g g^−1^, dry weight) of the muscle of *Mugil cephalus* and *Diapterus* spp. determined in samples collected in Sinaloa state, NW Mexico.

Zone	Species	Cd	Cu	Pb	Zn
Altata lagoon [5]	*M. cephalus *	0.6	6.3	—	—
Altata lagoon [6]	*M. cephalus *	0.3 ± 0.3		1 ± 0.3	18.4 ± 0.9
Coastal zone [7]	*M. cephalus *	0.04–0.47	1.18–1.24	0.81–2.4	
Urías lagoon [4]	*M. cephalus *	0.27–0.33	1.18–1.57	2.07–3.05	9.6–11.7
Fish markets*	*M. cephalus *	0.36 ± 0.01	2.40 ± 0.10	0.11 ± 0.09	19.7 ± 12.7
SE Sinaloa [4]	*Diapterus *spp.	0.25–0.33	0.21–1.86	0.89–4.91	8.41–15.3
Fish markets^∗/^	*Diapterus *spp.	0.28 ± 0.27	0.22 ± 0.12	0.04 ± 0.05	22.5 ± 9.5

*This study.
